# Surface Modification of Liposomes by a Lipopolymer Targeting Prostate Specific Membrane Antigen for Theranostic Delivery in Prostate Cancer

**DOI:** 10.3390/ma12050756

**Published:** 2019-03-05

**Authors:** Hooman Yari, Gregory Nkepang, Vibhudutta Awasthi

**Affiliations:** Department of Pharmaceutical Sciences, College of Pharmacy, University of Oklahoma Health Sciences Center, 1110 North Stonewall Avenue, Oklahoma City, OK 73117, USA; hyari@ouhsc.edu (H.Y.); gnkepang@ouhsc.edu (G.N.)

**Keywords:** prostate cancer, prostate specific membrane antigen (PSMA), liposome, targeted delivery, theranostic, nanoparticle

## Abstract

Prostate specific membrane antigen (PSMA) is a marker for diagnosis and targeted delivery of therapeutics to advanced/metastasized prostate cancer. We report a liposome-based system for theranostic delivery to PSMA-expressing (PSMA^+^) LNCaP cells. A lipopolymer (P^3^) comprising of PSMA ligand (PSMAL), polyethylene glycol (PEG_2000_), and palmitate was synthesized and post-inserted into the surface of preformed liposomes. These P^3^-liposomes were loaded with doxorubicin and radiolabeled with ^99m^Tc radionuclide to study their theranostic characteristics. Differential expression of PSMA on LNCaP and PC3 cells was confirmed by immunoblotting as well as by uptake of PSMAL labeled with ^18^F radionuclide. We found that the uptake of ^99m^Tc-labeled P^3^-liposomes by LNCaP cells was >3-fold higher than ^99m^Tc-labeled Plain-liposomes; the amount of doxorubicin delivered to LNCaP cells was also found to be >3-fold higher by P^3^-liposomes. Cell-based cytotoxicity assay results showed that doxorubicin-loaded P^3^-liposomes were significantly more toxic to LNCaP cells (*p* < 0.05), but not to PSMA-negative PC3 cells. Compared to doxorubicin-loaded Plain-liposomes, the IC_50_ value of doxorubicin-loaded P^3^-liposomes was reduced by ~5-fold in LNCaP cells. Together, these results suggest that surface functionalization of liposomes with small PSMA-binding motifs, such as PSMAL, can provide a viable platform for specific delivery of theranostics to PSMA^+^ prostate cancer.

## 1. Introduction

Prostate cancer is a leading cancer in men worldwide. According to the Cancer Statistics 2017, there were 161,360 new cases and 26,730 projected deaths attributed to prostate cancer [1]. Although the overall 5-year survival rate of prostate cancer patients in the USA is 97.3%, it declines drastically to 29% in patients with advanced stage prostate cancer [2,3]. Thus, early and accurate diagnosis of prostate cancer is vital to treatment success. The major challenge in management of prostate cancer is the treatment of castration-resistant prostate cancer and prostate cancer which has metastasized to the pelvic lymph nodes or other parts of the body. Primary diagnosis of prostate cancer is usually based on circulating levels of prostate-specific antigen combined with digital rectal exam, which help identify cancer at earliest stages in screening methodologies; ultrasound, biopsy, and magnetic resonance imaging are employed to confirm the initial screening results. 

Theranostics is a promising technology in which diagnostic and therapeutic capabilities are combined into a single agent and where the specificity of a diagnostic method is utilized to deliver drugs and monitor the kinetics of drug delivery [4]. An effective theranostic exploits a monitorable cancer-specific marker to guide drug delivery. For prostate cancer, prostate-specific membrane antigen (PSMA) has currently emerged as an attractive target for theranostic development to help individualize prostate cancer treatment and evaluate the effectiveness of treatment and cancer recurrence [5,6]. 

Prostate-specific membrane antigen is a 100 kDa type II carboxypeptidase transmembrane protein with a large extracellular domain [7]. Also called folate hydrolase 1, it is highly over-expressed in prostate cancer cells, including castration-resistant prostate cancer, even when staining for prostate-specific antigen is negative; it is transferred to the luminal surface of the prostatic ducts upon malignant transformation [5,8,9]. It is also expressed in other organs such as kidney, proximal small intestine, and salivary glands; however, expression of PSMA in prostate cancer is about a thousand-fold higher than in these other tissues [7,10]. The discovery that PSMA can bind to glutamate-containing molecules has led to design and synthesis of glutamate-like PSMA inhibitors that contain motifs mimicking the substrate or the hydrolysis reaction intermediates [7,11,12,13]. One major class of these compounds contains a lysine–urea–glutamine (Lys–urea–Glu) motif that binds to PSMA protein with high affinity and specificity [14,15,16]. These compounds have been previously used for targeted delivery of radionuclides for imaging and therapy of prostate cancer [17,18,19]. 

We hypothesize that PSMA-targeted delivery of chemotherapy can enhance therapeutic efficacy by reducing the required therapeutic dose and preventing the toxicity associated with non-target effects of anticancer drugs. The objective of this study was to develop theranostic liposomes loaded with doxorubicin, which are targeted to PSMA by surface modification with a PSMA-ligand (PSMAL). Liposomes are versatile drug carriers that can favorably alter pharmacokinetics and distribution of loaded drugs; they are known to readily distribute in tumor tissue due to decreased endothelial integrity in tumor vasculature. Besides the widespread use of already approved liposomal doxorubicin (Doxil^®^) and liposomal daunorubicin (DaunoXome^®^) in oncology, a docetaxel-loaded liposome preparation has also shown success in a prostate cancer clinical trial [20]. Our previous work has demonstrated the potential of liposomes for surface modification [21,22], which has been employed for ligand conjugation and targeting. In this article, we present the preparation and evaluation of doxorubicin-containing PSMAL-liposomes which could be radiolabeled with a gamma-emitting radionuclide ^99m^Tc for real-time monitoring with non-invasive gamma camera imaging. 

## 2. Materials and Methods

### 2.1. Materials

Mouse monoclonal anti-human PSMA antibody (PSM-F2) and mouse immunoglobulin G kappa (IgGĸ)-binding protein (BP) conjugated to horseradish peroxidase (m-IgGκ-BP-HRP) were purchased from Santa Cruz Biotechnology (Dallas, TX, USA). Alexa Fluor® 488 anti-human PSMA antibody and Alexa Fluor^®^ 488 mouse IgG1ĸ were purchased from Biolegend (San Diego, CA, USA). Dipalmitoylphosphatidylcholine (DPPC) and dimyrsitoylphosphatidylglycerol (DMPG) were purchased from NOF Corporation (Tokyo, Japan). Distearoylphosphatidylethanolamine- methoxy(polyethylene glycol)-2000 (DSPE-PEG_2000_) was acquired from Avanti Polar Lipids (Alabaster, AL, USA). Highly-purified cholesterol was purchased from Calbiochem (San Diego, CA, USA). Doxorubicin hydrochloride was obtained from LC Laboratories (Woburn, MA, USA). For radiolabeling, ^99m^Tc was provided as Na^99m^TcO_4_ by OUHSC-Nuclear Pharmacy (Oklahoma City, OK, USA). Hexamethylpropyleneamine oxime (d,l-HMPAO) was synthesized in-house and made into a kit by modifications of a method reported elsewhere [23]. Recombinant histidine(His)-tagged human PSMA protein was purchased from LifeSpan BioSciences, Inc. (Seattle, WA, USA). Cell culture media and supplements were purchased from Gibco-Thermo Fisher Scientific (Waltham, MA, USA). Human prostate cancer cell lines PC3 (ATCC CRL-1435) and LNCaP clone FGC (ATCC CRL-1740) were obtained from American Type Culture Collection (Manassas, VA, USA). All other chemicals were obtained from various suppliers represented by VWR Scientific (West Chester, PA, USA) or from Sigma–Aldrich (St. Louis, MO, USA).

### 2.2. Cell Culture

PC3 cells were cultured in high-glucose Dulbecco’s Modified Eagle medium supplemented with 10% heat-inactivated fetal bovine serum (FBS), Na-pyruvate (1 mM), and 1% of penicillin–streptomycin cocktail (Invitrogen, Carlsbad, CA, USA) and were maintained at 37 °C in 5% CO_2_ atmosphere. LNCaP cells were cultured similarly in Roswell Park Memorial Institute-1640 medium supplemented with 10% FBS.

### 2.3. Western Blotting

Membrane expression of PSMA protein in LNCaP and PC3 cells was determined by Western blot analysis. Membrane preparations were prepared by a method published elsewhere [10]. Briefly, LNCaP and PC3 cells were washed twice and scraped into ice-cold phosphate buffered saline (PBS, pH 7.4). Cells were spun at 1000× *g* for 5 min at 4 °C and 1 mL of ice-cold 1 mM NaCO_3_ containing protease inhibitor cocktail (Mammalian ProteaseArrest; G-BioSciences, St. Louis, MO, USA) was added to the pellet. After incubation on ice for 30 min, cells were homogenized and the homogenates were centrifuged at 2000× *g* for 5 min at 4 °C. Supernatant was collected and centrifuged at 137,000× *g* in an Optima L-100 XP ultracentrifuge (Beckman Coulter, Brea, USA) for 2 h at 4 °C. Pelleted membrane fraction was suspended in PBS containing protease inhibitors. Protein concentration was determined by the bicinchoninic acid (BCA) assay kit (Thermo Fisher Scientific, Richardson, TX, USA).

Membrane preparations were separated on a 10% denaturing polyacrylamide gel and transferred onto nitrocellulose membranes by using Trans-Blot Turbo transfer system (Bio-Rad, Hercules, CA, USA). The transfer membranes were blocked by 1% bovine serum albumin in PBS and probed with primary anti-human-PSMA antibody, followed by secondary m-IgGκ-BP-HRP. Protein bands were visualized in a FlourChem FC2 imaging system (Cell Bioscience, Santa Clara, CA, USA) by applying SuperSignal West Pico chemiluminescent substrate (Thermo Fisher Scientific, Richardson, TX, USA). To verify equal loading of proteins, membranes were stripped in a buffer comprising of 0.2 M glycine (pH 2.2), 0.1% w/v sodium dodecyl sulfate, and 1% Tween 20, followed by staining with Ponceau S solution.

### 2.4. Flow Cytometry

Surface expression of PSMA in LNCaP and PC3 cells was also determined by flow cytometry. Briefly, 1 × 10^6^ cells were washed with a cell-staining buffer (Biolegend, San Diego, CA, USA) and blocked in 1% bovine serum albumin in cell-staining buffer. Subsequently, cells were stained with Alexa Fluor^®^ 488 anti-human PSMA antibody or Alexa Fluor^®^ 488 mouse IgG1 as the isotype control. Unstained cells were processed in the same manner with no agent added to the cell-staining buffer. The cells were read on a Stratedigm S1400Exi system (Stratedigm Inc., San Jose, CA, USA).

### 2.5. Synthesis of PSMA Ligand (PSMAL)

**Scheme 1:**
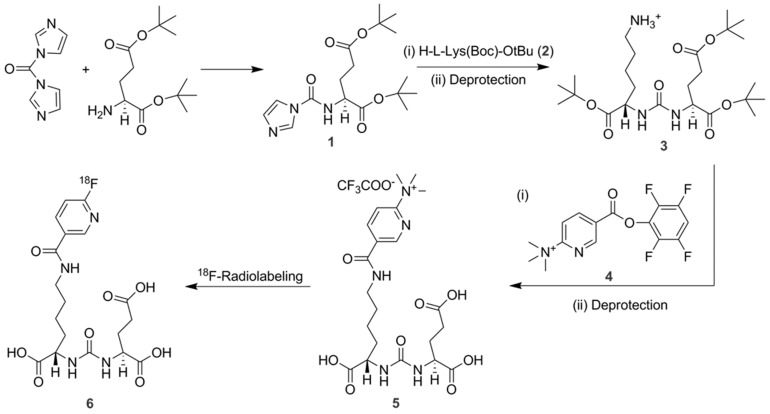
Synthesis scheme for prostate specific membrane antigen-ligand (PSMAL, **5**) and ^18^F-radiolabeled PSMAL (**6**).

Compound **5** (PSMAL, Scheme 1) is an intermediate to synthesize our compound of interest P^3^. It was synthesized in several steps as follows. Compound **1** was synthesized according to the method described elsewhere [19]. To a solution of **1** (1.5 g, 5.045 mmol) in dichloroethane at 0 °C was added methyl triflate (0.56 µL, 5.1 mmol) and triethylamine (1.35 mL, 10.11 mmol). The mixture was stirred at 0 °C for 70 min. This was followed by addition of **2** and the reaction was maintained at 0 °C for additional 20 min, before letting the temperature rise to 40 °C over 4 h. The reaction mixture was diluted with dichloromethane and sequentially washed with saturated NaHCO_3_, saturated NaCl, and water (2 × 100 mL each). Silica gel column chromatography using ethyl acetate/hexane (1:1) afforded the protected product as yellow oil (2.74 g, 80% yield). Calculated mass for C_29_H_53_N_3_O_4_: 587.3782; observed mass for (M + Na): 610.397. Deprotection of tert-butyloxycarbonyl group using 1 M HCl in ethyl acetate afforded compound **3** which was used without further purification in the next steps (calculated mass for C_24_H_46_N_3_O_7_: 487.2753; observed for (M + H): 488.2753).

In 6 mL of anhydrous dichloromethane, compound **3** (409.4 mg, 0.78 mmol) was dissolved, followed by addition of triethylamine (216.9 µL, 1.62 mmol). After stirring the mixture for 20 min, compound **4** (374.0 mg, 0.78 mmol) was added and the reaction mixture was further stirred overnight at room temperature; compound **4** was synthesized separately from a 2-step synthesis as described elsewhere [24]. The reaction mixture was diluted with 100 mL dichloromethane and washed three times with water. Organic layer afforded the protected compound as brown oil (650 mg, ~80% yield). Calculated mass for C_33_H_56_N_5_O_8_^+^: 650.4123; observed mass for M: 650.3294. Deprotected compound **5** was obtained by treatment with 100% trifluoroacetic acid for 5 h at room temperature. Trifluoroacetic acid was removed using nitrogen gas at room temperature. Compound **5** was purified using acetonitrile/H_2_O (10:90 v/v) mixture as solvent on a C18 cartridge. Ultraviolet-reactive fractions were pooled, and solvent was evaporated to afford compound **5** (PSMAL) as pale-yellow oil. Calculated mass for C_21_H_32_N_5_O_8_^+^: 482.2245; observed mass: 482.2291.

### 2.6. ^18^F-Radiolabeling of PSMAL

Briefly, [^18^F]F^−^ was produced by irradiating enriched [^18^O]H_2_O with proton beam in a Biomarker Generator (ABT Molecular Imaging, Louisville, TN, USA) as described previously [25]. Aqueous [^18^F]F^−^ (560 µL) was transferred to a reaction vial containing 8 mg tetrabutyl ammonium bicarbonate. Solvent was evaporated by acetonitrile-mediated azeotropic drying under nitrogen stream (2.7–3.0 psi) at 100 °C. To the dried residue, approximately 2 mg of PSMAL (**5**) was added as a solution in 700 µL of dimethyl formamide/acetonitrile mixture (2:5 v/v). The reaction mixture was heated in a sealed vial at 60 °C for 15 min, followed by solvent removal. The vial contents were mixed in 1.5 mL of high-performance liquid chromatography (HPLC)-grade water and passed through a 5 µm nylon filter. The filtered solution was injected into an Azura® HPLC system (Knauer, Berlin, Germany) for purification of [^18^F]-PSMAL (compound **6**, Scheme 1). A mixture of 18% ethanol in 0.2% phosphoric acid was used as mobile phase. A fraction from 19 to 22 min was collected as [^18^F]-PSMAL and its tonicity (290 mOsm/L) and pH (6.0–6.5) were adjusted. 

**Scheme 2:**
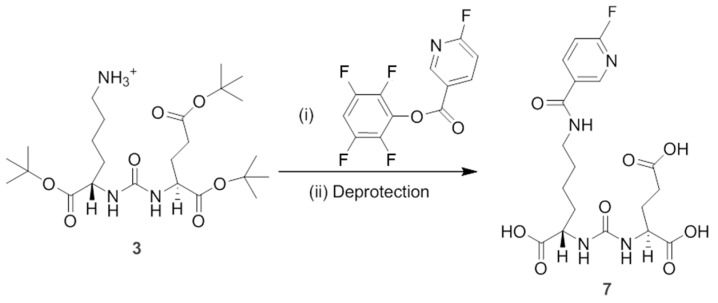
Synthesis scheme for unlabeled-PSMAL (^19^F-PSMAL; compound **7**).

To synthesize non-radioactive PSMAL as standard (compound **7** containing ^19^F), we used Scheme 2. Briefly, compound **3** (150 mg, 0.29 mmol) was dissolved in 5 mL dry dichloromethane and 79.46 µL of triethylamine was added (1.43 mmol). After mixing for 20 min at room temperature, equimolar amount of pentahydroxybenzene ester of 6-fluoronicotinic acid was added and the reaction was allowed to occur at room temperature for 5 h. The solvent was evaporated *in vacuo* and the product was purified by silica gel column chromatography using ethyl acetate/hexane solvent gradient. Starting with ethyl acetate/hexane (25:75 v/v), the solvent polarity was increased gradually to 70:30. Fractions were monitored by instant thin-layer chromatography (TLC) and UV-fluorescence to afford the protected intermediate product as colorless oil (92 mg, 75% yield; calculated mass for C_30_H_47_FN_4_O_8_Na (M + Na): 633.3276; observed for (M + Na): 633.3222). This intermediate compound was deprotected by using 2 mL of trifluoroacetic acid for 3 h; the mixture was dried under nitrogen flow at room temperature, and compound **7** was purified by HPLC using 15% acetonitrile in water as solvent. Calculated mass for C_18_H_23_FN_4_O_8_Na (M + Na): 465.1398; observed mass for (M + Na): 465.1429.

### 2.7. Synthesis of Palmitate-PEG_2000_-PSMA (P^3^)

**Scheme 3:**
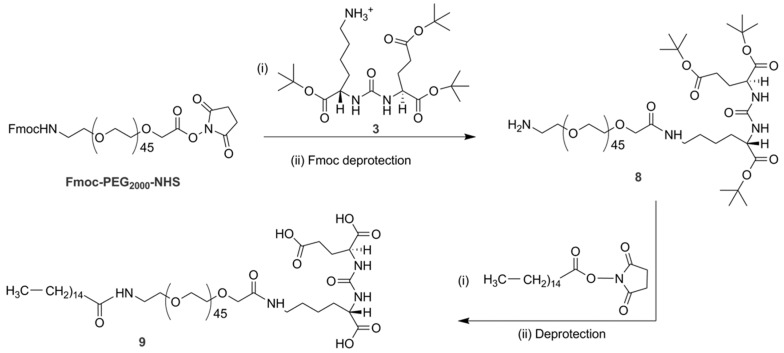
Synthesis scheme for lipopolymer P^3^ (compound **9**).

The desired lipopolymer P^3^ (compound **9**, Scheme 3) was synthesized in two major steps. 

Synthesis of NH_2_-PEG_2000_-PSMA-tert-butyl esters (compound **8**): To a solution of compound **3** (156 mg, 0.3 mmol) in anhydrous dimethyl formamide (5 mL), excess triethylamine (68 µL, 1.2 mmol) was added and the mixture was allowed to stir for 10 min. Commercially available fluorenylmethyloxycarbonyl (Fmoc)-protected PEG_2000_-NHS (400 mg, 0.2 mmol) was added and the reaction mixture was stirred at room temperature for 24 h. The crude reaction mixture was passed through a G-15 Sephadex column using dimethyl formamide as solvent. The fast-moving spot at the solvent front was collected as the product. ^1^H-NMR (CD_3_OD, 300 MHz): δ-8.003 (N-H, S, 2H), 7.83 (=C-H, d, 2H), 7.69 (=C-H, d, 2H), 7.45–7.32 (=C-H, m, 4H), 4.39 (CH_2_, d, 4H), 4.15 (CH, m, 4H), 4.00 (CH_2_, S, 2H), 3.62 (CH_2_)_n_[PEG], 3.36–3.21 (m, CH2) 1.91–1.55 (m, CH_2_) 1.39 (CH_3_, d, 27H) 1.31–1.26 (m, CH_2_).

Fmoc protection was removed by adding 20% (v/v) of piperidine into a dimethyl formamide solution of the collected product and stirring at room temperature for 3 h. The deprotected product was dialyzed (MWCO 1000 Da) for 24 h against dimethyl formamide, followed by dialysis against dichloromethane for 24 h to remove dimethyl formamide. The product in dichloromethane was concentrated *in vacuo* to afford a brown oily product (321 mg, ~70%). 

Synthesis of compound **9** (P^3^): Compound **8** (350 mg, 0.15 mmol) was dissolved in vigorously- stirred dry dimethyl formamide (5 mL). Triethylamine (51 µL, 6 equivalents) was added, followed by addition of *N*-hydroxysuccinimide-palmitate (64.57 mg, 0.183 mmol). The reaction was allowed to occur at room temperature for 36 h. The crude product was precipitated in cold diethyl ether to remove unreacted palmitic acid, followed by separation on G-15 Sephadex column using methanol as solvent; fast moving spot was collected and dialyzed against water for 24 h. Compound **9** was obtained as a brown product by deprotecting the intermediate compound using TFA and drying *in vacuo*. ^1^H-NMR (CD_3_OD, 300 MHz): 7.95 (N-H, S, 2H), 4.39 (CH_2_, d, 4H), 4.15 (CH, m, 4H), 4.00 (CH_2_, S, 2H), 3.62 (CH_2_)_n_[PEG], 3.36–3.21 (CH_2_, m,) 1.91–1.55 (CH_2_, m,) 1.31–1.25 (m, CH_2_), 0.85 (CH_3_, S, 3H).

### 2.8. ^18^F-PSMAL Uptake Assay

LNCaP and PC3 cells (1 × 10^5^) were seeded in 24-well poly-D-lysine-coated plates and allowed to grow for 72 h and reach 80–90% confluence. Medium in the wells was replaced with 300 µL of radioactive medium containing ^18^F-PSMAL (4 µCi/well). After 1 h incubation at 37 °C, the medium was removed, and the cells were washed three times with PBS. Cells were lysed in 300 µL of radioimmunoprecipitation assay (RIPA) buffer and incubation for 10 min at 37 °C. Radioactivity counts were measured in cell lysates using a Packard Cobra II Auto gamma counter (Perkin Elmer, Waltham, MA, USA). In competition assay, ^18^F-PSMAL uptake was determined in presence of excess concentrations of unlabeled PSMAL (compound **7**; 0.5, 1, and 2 µM) or P^3^ (compound **9**; 0.5 and 1 µM). Uptake value was calculated as counts per min (CPM) per mL of lysate. 

### 2.9. Critical Micellar Concentration (CMC)

The CMC of P^3^ was determined by a fluorescence-based method as described elsewhere [22,26]. Briefly, stock solution of *N*-phenyl-1-naphthylamine (NPN, 1 mM in absolute ethanol) was diluted with PBS to 1 µM working concentration. Increasing amounts of P^3^ solution in PBS (3.2 mM) were added to the NPN 1 µM solution to obtain a series of concentrations ranging from 0 to 80 µM of P^3^. The mixtures were incubated for 30 min at room temperature. A 5000U-DR15 spectrofluorometer (Shimadzu, Columbia, MD, USA) equipped with a Xenon excitation source was used to excite samples at 340 nm and fluorescence was recorded from 360 to 520 nm at 90° detection angle. The corresponding shifts in the maximum wavelength of the emission spectrum of NPN vs. P^3^ concentration were plotted to find an inflection point indicative of the CMC value of P^3^.

### 2.10. Liposome Preparation

Liposomes capable of radiolabeling with ^99m^Tc radionuclide were made using a method described elsewhere [27,28]. The lipid composition of DPPC (247 mg, 336.5 µmol), DMPG (51 mg, 74 µmol), cholesterol (129 mg, 333 µmol), and vitamin E (0.645 mg, 1.5 µmol) in 45:10:44.8:0.2 mol% was dissolved in a chloroform–methanol mixture (2:1). Solvent was evaporated on a Buchi R-210 rotary evaporator (Flawil, Switzerland) to form a thin lipid film. The lipid film was kept overnight under vacuum to remove any residual organic solvent. The dried lipid film was rehydrated in 10 mM sucrose and the suspension was lyophilized in a Triad freeze-dryer (Labconco, Kansas City, MO, USA). The lyophilized material was rehydrated with glutathione solution in water (100 mM, pH 6.5), followed by sequential extrusion at 45 °C through polycarbonate membranes of 1, 0.6, 0.4, and 0.2 µm pore size (Lipex Biomembranes Inc., Vancouver, Canada); each extrusion was repeated five times. The extruded preparation was centrifuged at 45,000 rpm (244,717× *g*) for 30 min at 4 °C by using an Optima L-100 XP Ultracentrifuge (Beckman Coulter, Fullerton, California, USA). The liposome pellet was washed with PBS to remove the majority of extra-liposomal glutathione and suspended in PBS. Glutathione-containing ammonium sulfate-gradient liposomes suitable for remote loading of doxorubicin were also similarly prepared. The remote loading of doxorubicin is described below in Section 2.15.

Hydrodynamic diameter and zeta potential of liposomes were determined by dynamic light scattering (DLS) technique using a ZetaPALS analyzer (Brookhaven Instruments, Holtsville, NY, USA). Size and charge were measured by diluting 10 µL liposome in 3 mL of PBS (pH 7.4). Phospholipid concentration in liposome suspensions was determined by a colorimetric method described elsewhere [29]. 

### 2.11. Surface Modification of Liposomes by Post-insertion of P^3^

For post-insertion of P^3^, 0.5 mL of liposome suspension was diluted with PBS to 25 mL (DPPC = 3.4 mM). Amount of P^3^ equal to 10 mol% of DPPC was dissolved in 50 mL of PBS and filtered through a 0.2 µm sterile filter. The P^3^ solution was injected into the diluted liposome suspension at a rate of 50 µL/min by using a syringe pump (Chymex Inc., Stafford, TX, USA) with moderate stirring at room temperature (25–27 °C). After further incubation for 1 h at room temperature, the suspension was centrifuged at 45,000 rpm (244,717× *g*) for 30 min at 4 °C. The liposome pellet was resuspended and diluted in PBS and re-centrifuged to remove any residual free or micellar P^3^. After three wash-cycles, the pellet was resuspended in 300 µL of 300 mM sucrose in PBS. Each time, a portion of original liposome without post-insertion was kept for use as non-targeted control liposomes.

### 2.12. Radiolabeling of Liposomes with ^99m^Tc

Liposomes were labeled with ^99m^Tc by a method described previously [30]. Briefly, a lipophilic complex of ^99m^Tc with HMPAO was formed by adding ^99m^Tc-pertechnetate and allowing 5 min incubation at room temperature. Formation of lipophilic ^99m^Tc-HMPAO complex, free ^99m^Tc-pertechnetate, and reduced/hydrolyzed ^99m^Tc was monitored by TLC to ensure ≥80% of lipophilic complex. The details of this TLC method and result analysis are described elsewhere [23]. This freshly prepared ^99m^Tc-HMPAO complex was mixed with a liposome preparation in equal volumes and the mixture was incubated at room temperature for 30 min with occasional swirling. To remove extra-vesicular ^99m^Tc-HMPAO, the liposome mixture was loaded on a PD-10 column (GE Healthcare Sciences, Pittsburg, PA, USA) and eluted with PBS. To verify the absence of free ^99m^Tc in radiolabeled liposome preparations, TLC in saline as a mobile phase was used. In this system, free ^99m^Tc and ^99m^Tc-HMPAO travelled with the solvent front, whereas ^99m^Tc-liposomes remained at the origin. To determine radiolabeling efficiency, radioactivity of liposome suspensions was measured before and after purification over PD-10 columns.

(1) $$\mathrm{Labeling}\;\mathrm{efficiency}=\frac{\mathrm{Post}-\mathrm{column}\;\mathrm{radioactivity}}{\mathrm{Pre}-\mathrm{column}\;\mathrm{radioactivity}}\times 100$$

### 2.13. Binding of ^99m^Tc-P^3^-Liposomes to PSMA Protein

We prepared PSMA-bound columns to study the binding of P^3^-liposomes. HisPur Ni–NTA spin columns (Thermo Scientific, Rockford, IL, USA) were equilibrated at 4 °C and washed with 400 µL of a buffer (imidazole 20 mM in PBS, pH 7.4). The columns were forced-eluted by centrifugation at 700× *g* for 2 min. Approximately 1 µg histidine-tagged human PSMA protein (in 400 µL of equilibrium buffer) was cycled 5 times through the column. After washing the column, 400 µL of radiolabeled liposome preparation was similarly cycled through the column. ^99m^Tc-labeled Plain-liposomes and ^99m^Tc-labeled P^3^-liposomes were diluted in the equilibrium buffer to a radioactivity concentration of 25 µCi/mL. The columns were washed 5 times with 400 µL of wash buffer (imidazole 25 mM in PBS, pH 7.4). The column-bound ^99m^Tc-liposomes were eluted with 400 µL of the elution buffer (imidazole 250 mM in PBS, pH 7.4). The eluted fractions were counted for radioactivity concentration in a gamma counter.

### 2.14. Cellular Uptake of ^99m^Tc-P^3^-Liposomes

LNCaP cells (2 × 10^5^) were seeded in a poly-D-lysine-coated 24-well plate and allowed to grow for 48 h. The culture medium was replaced with 200 µL medium containing ^99m^Tc-Plain-liposomes and ^99m^Tc-P^3^-liposomes (20 µCi/mL). For competition assay, the wells were pre-incubated with 200 µL of P^3^ solution (compound **9**; 100 µM in medium) for 10 min prior to treatment with radiolabeled liposomes. After 1 h incubation at 37 °C, the medium was removed and the cells were washed three times with 500 µL of PBS. The washed cells were lysed by adding RIPA buffer (300 µL) and incubating for 10 min at 37 °C. The rest of the processing to determine cell-associated radioactivity was identical to that described above in Section 2.8. To account for binding of ^99m^Tc-liposomes to the surface of the plate, cell-free wells were treated in the same manner.

### 2.15. Intracellular Delivery of Doxorubicin by P^3^-Liposomes in LNCaP Cells

In order to study the delivery of doxorubicin by liposomes and its cytotoxicity, we loaded doxorubicin in ammonium sulfate-gradient liposomes as described in our previous article [28]. Briefly, the external phase of liposome suspensions was replaced by 10 mM HEPES-buffered saline (HBS, pH 7.4) by ultracentrifugation at 45,000 rpm (244,717× *g*). Liposomes were diluted with HBS to phospholipid concentration of 1.6 mM and doxorubicin∙HCl solution in HBS (1 mM) was added to maintain the drug-to-phospholipid ratio at 1 mmol:10 mmol. The mixture was kept for 24 h with moderate swirling, and final doxorubicin-loaded liposomes were separated by ultracentrifugation. The liposomes were washed 3 times with PBS to remove unentrapped doxorubicin from liposome suspensions and the final pellet was suspended in 300 mM sucrose in PBS (pH 6.2). Doxorubicin concentration in liposome suspensions was determined by using a colorimetric assay [28,31]. Briefly, the liposomes were lysed in aqueous Triton X-100 solution (0.5% v/v) and absorption was measured at 480 nm by using a Lambda 4B UV/Vis spectrophotometer (Perkin-Elmer Inc., Waltham, MA, USA). 

Intracellular delivery of doxorubicin by P^3^- and Plain-liposomes was quantified by measuring the intensity of fluorescence light emitted by doxorubicin internalized in LNCaP cells [32]. LNCaP cells were detached from culture flasks by using 3 mM ethylene diamine tetraacetic acid (EDTA) solution and the cell suspension (2 × 10^5^ cells) was washed with 1 mL of cold PBS followed by spinning at 200× *g* for 5 min at 4 °C. Cell pellets were re-suspended in 500 µL of doxorubicin-loaded P^3^- or Plain-liposomes in PBS (doxorubicin concentration 40 µM). Cells in control tubes were treated only with PBS in the same manner. After 30 min incubation on a rocking platform at 4 °C, cells were centrifuged, and washed with PBS to remove extracellular liposomes. Finally, the cells were lysed in 5% Triton X-100-PBS. Fluorescence was measured at excitation wavelength 460 nm and emission wavelength 590 nm by a Synergy 2 plate reader (Biotek, Winooski, VT, USA). 

### 2.16. Cytotoxicity of Doxorubicin-Loaded Liposomes

Immortalized LNCaP and PC3 cells were seeded in 96-well plates at a density of 3000 cells/well. After 24 h, the cells were treated with doxorubicin-loaded P^3^-liposomes or doxorubicin-loaded Plain-liposomes in fresh medium (doxorubicin concentration 1 µM). Drug-containing medium in the wells was replaced with drug-free medium at 0.5, 1, 2, 4, and 6 h and the cells were further incubated to a total duration of 48 h. In additional set of wells, the medium was not changed and the cells were allowed drug exposure continuously for 48 h duration. Cell viability was determined by 3-(4,5-dimethylthiazol-2-yl)-5-(3-carboxymethoxyphenyl)-2-(4-sulfophenyl)-2*H*-tetrazolium (MTS) reagent as described elsewhere [33]. Absorption was read at 490 nm on Synergy 2 plate reader. To determine IC_50_ values of doxorubicin-loaded liposomes, a similar treatment procedure was used; doxorubicin concentration in IC_50_ experiments was in the range of 10 to 0.001 µM. 

For additional comparison, we also studied toxicity of drug-less Plain- and P^3^-liposomes in LNCaP cells. The phospholipid concentration in treatments was maintained at the same level as was used with drug-loaded Plain- and P^3^-liposomes. 

### 2.17. Data Analysis

The data were analyzed by using GraphPad Prism for Windows Ver. 7.03 software (La Jolla, CA, USA). Average values are given as mean ± standard error of the mean (SEM) of minimum of three replicates. For comparison of mean values, two-tailed *t*-test and analysis of variance (ANOVA) with Tukey correction were used for single- or multiple comparisons, respectively. *p*-value < 0.05 was considered acceptable for statistical significance. Radioactivity calculations were background-subtracted and corrected for decay as per radionuclide decay half-life (^18^F = 110 min and ^99m^Tc = 6 h). 

## 3. Results and Discussion

For prostate cancer targeting, PSMA is among the most common molecular targets which also include gastrin-releasing peptide receptor, urokinase receptor, folate receptor, and sigma receptor [34,35,36,37,38]. It is also known as *N*-acetyl-α-linked acidic dipeptidase in the central nervous system and folate hydrolase 1 in the intestine. In prostate cancer, PSMA has been explored as a target for therapeutic delivery and diagnosis using nanoparticles; major strategies for PSMA-targeting include PSMA-specific antibodies and aptamers, folate-modification, and conjugation with synthetic PSMA ligands. In recent times, research on Lys–urea–Glu motif to develop PSMA-targeted nanocarriers has attracted significant attention [34,39,40]. This targeting strategy using a small molecule ligand does not suffer from complications associated with large molecule ligands (antibodies), complex chemistry, and processing. Antibody-lipid complexes may also cause problems associated with aggregation of liposomes, Fc-receptor binding, complement activation, and immunogenicity [41,42,43]. Moreover, unlike other approaches mentioned above, compounds containing Lys–urea–Glu motif are already in clinic for diagnostic imaging purposes [18]. However, PubMed literature searches to date, using keywords “prostate cancer”, “PSMA”, and “liposome”, showed no reports on liposomes modified with Lys–urea–Glu motif for therapeutic or diagnostic purposes. In this work, we report PSMA-targeted liposomes and evaluate their potential to serve as a theranostic preparation.

Liposomes are recognized as a most attractive nanocarrier because of several well-known advantages, especially related to the ease with which both water-soluble and lipid-soluble drugs can be loaded and the ability to modify their surface for targeting and stealth purposes [22,28,44]. Recently, uptake of folate-modified liposomes loaded with doxorubicin and mitomycin was found to improve 8- to 10-fold in prostate cancer [45]. Others have used liposomes to target prostate cancer primarily by conjugation with an antibody [46,47,48,49]. Liposomes have also been modified with a PSMA-specific aptamer to deliver a pro-oxidant Zn-chelator *N*,*N*,*N*′,*N*′-tetrakis(2-pyridylmethyl)-ethylenediamine [50]. More recently, Wang et al. showed targeting of α-enolase, which is overexpressed in prostate cancer, by using liposomes conjugated with a peptide ligand pHCT74 [51].

### 3.1. PSMA Expression in Prostate Cancer Cell Lines

**Figure 1:**
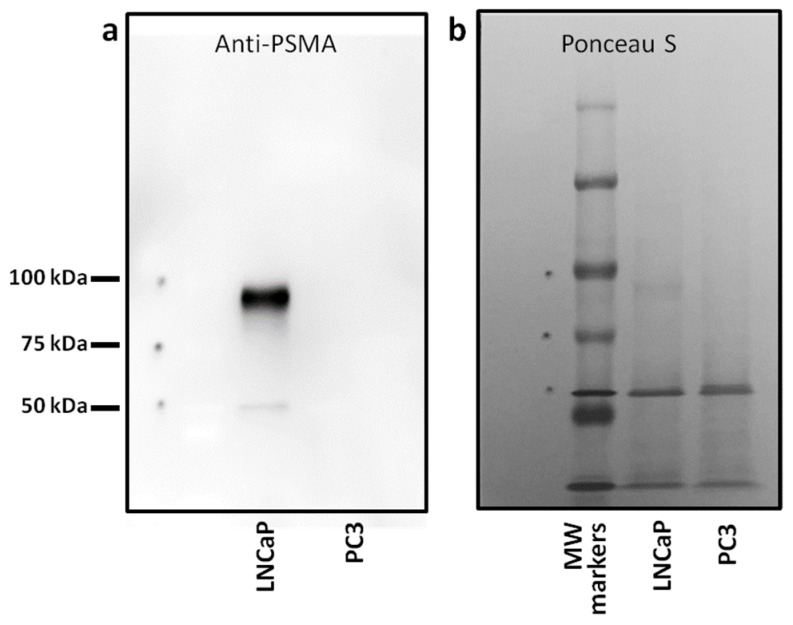
Western blots of prostate specific membrane antigen (PSMA) protein in membrane fractions isolated from LNCaP and PC3 cells. (**a**) Membrane fractions were immunoblotted using anti-PSMA antibody. (**b**) The same membrane was stained with Ponceau S stain.

**Figure 2:**
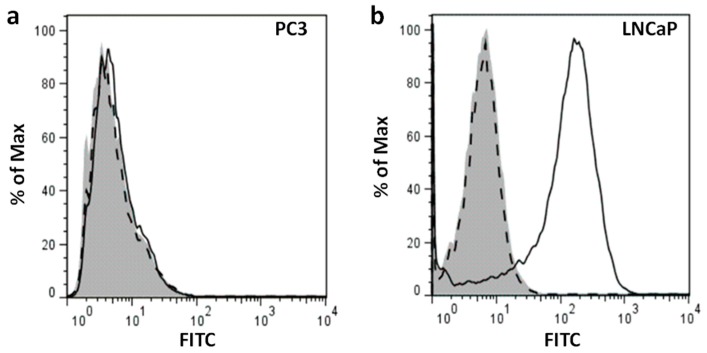
Flow cytometry of PSMA expression in (**a**) PC3 and (**b**) LNCaP cells. Anti-human PSMA antibody conjugated to Alexa Fluor® 488 and corresponding isotype control were used to stain the cells. Unstained cells are shown in gray. Dashed lines indicate the isotype control and solid lines show PSMA-positive cells.

The primary objective of this study was to create liposomes that are modified with a PSMA-binding small molecular probe to target prostate cancer cells. We chose LNCaP and PC3 human prostate cancer cell lines to test our liposomes. First, we established the expression of PSMA protein in our test cell line (LNCaP) and control cell line (PC3) by Western blotting and flow cytometry. Immunoblotting of membrane preparation showed a predominant 100 kDa band immunoreactive with anti-PSMA antibody in LNCaP cells; corresponding immunoblot of PC3 cells did not show this band (Figure 1a). Ponceau staining of the blots showed equal amounts of protein per lane (Figure 1b). Flow cytometry also showed cell surface expression of PSMA in intact LNCaP cells (Figure 2b); no significant fluorescence signal was observed in PC3 cells (Figure 2a). Together, these results suggest high expression of PSMA protein on the membrane of LNCaP cells and lack of PSMA expression in PC3 cells. Thus, LNCaP and PC3 are suitable cell lines to be used as PSMA^+^ and PSMA^−^ models in our study, respectively. Other reports are in agreement with our observation that LNCaP cells overexpress PSMA on their surface whereas PC3 cells lack PSMA expression [52,53]. 

### 3.2. Ligand PSMAL Binds to PSMA-Expressing LNCaP Cells

Glutamate-containing molecules such as *N*-acetyl-l-aspartyl-l-glutamate in the brain or folyl-poly-γ-glutamate in the intestine are hydrolyzed by PSMA protein [7,11,12]. Both enzymatic activities require binding of PSMA to glutamate residues, which has been exploited to design glutamate-like PSMA inhibitors containing motifs that mimic PSMA substrates or the hydrolysis reaction intermediates [13]. These inhibitors often include a glutamate residue which binds to the S1’ pocket of PSMA protein, and a urea bond that mimics the peptide bond or a phosphonate or phosphoamidate functional group mimicking transitional state in the hydrolysis reaction [14,15,16]. Compounds containing Lys–urea–Glu motif have been used in designing radiotracers for prostate cancer imaging [17,18,19]. In this study, we synthesized compound **5** (PSMAL) that contained Lys–urea–Glu motif for binding to PSMA (Scheme 1). An ^18^F-labeled version of PSMAL is known as 2-(3-(1-carboxy-5-[(6-[^18^F]fluoro-pyridine-3-carbonyl)-amino]-pentyl)-ureido)-pentanedioic acid ([^18^F]DCFPyL) in the literature [18].

**Figure 3:**
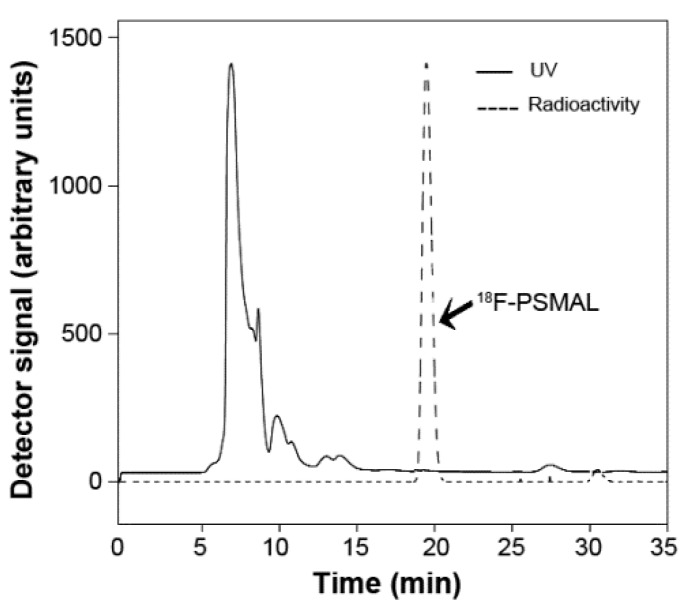
A representative radio-chromatogram of ^18^F-labeled PSMA ligand (^18^F-PSMAL; compound **6**) is shown. The UV absorption was measured at 250 nm.

**Figure 4:**
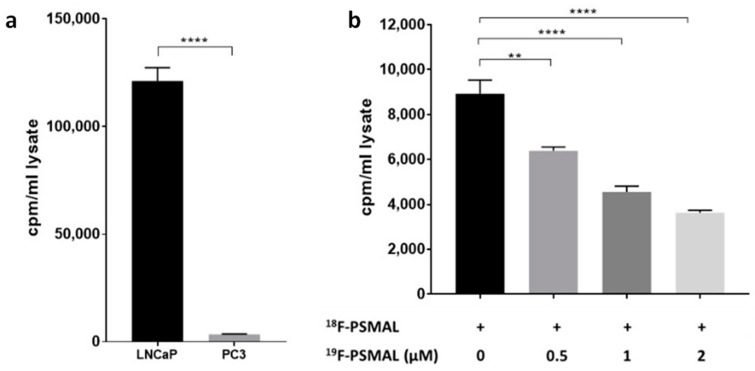
(**a**) Uptake of ^18^F-radiolabeled PSMAL (^18^F-PSMAL) in LNCaP and PC3 cells. (**b**) Interaction of ^18^F-PSMAL with LNCaP cells in the presence of unlabeled PSMAL (^19^F-PSMAL; compound **7**) (** *p* = 0.0021, **** *p* < 0.0001).

To examine that PSMAL interacts with cell surface PSMA protein, we exposed LNCaP cells to ^18^F-labeled PSMAL synthesized in-house. On the day of experiment, we synthesized ^18^F-PSMAL (compound **6**) by nucleophilic substitution in precursor compound **5**; the decay uncorrected radiochemical yield was 27.95 ± 5%. A representative HPLC radio-chromatogram of ^18^F-PSMAL is shown in Figure 3. Incubation of LNCaP with tracer amounts ^18^F-PSMAL for 1 h showed that the uptake of this ligand was significantly higher in PSMA^+^ LNCaP cells (Figure 4a). In PSMA^−^ PC3 cells, there was negligible uptake of ^18^F-PSMAL (Figure 4a). When the uptake of ^18^F-PSMAL in LNCaP cells was challenged by excess of non-radioactive PSMAL (compound **7**, ^19^F-PSMAL), the binding of ^18^F-PSMAL was significantly and dose-dependently reduced (Figure 4b), which indicates specificity of interaction between the ligand and its target. 

### 3.3. Lipopolymer P^3^ Competes with ^18^F-PSMAL

**Figure 5:**
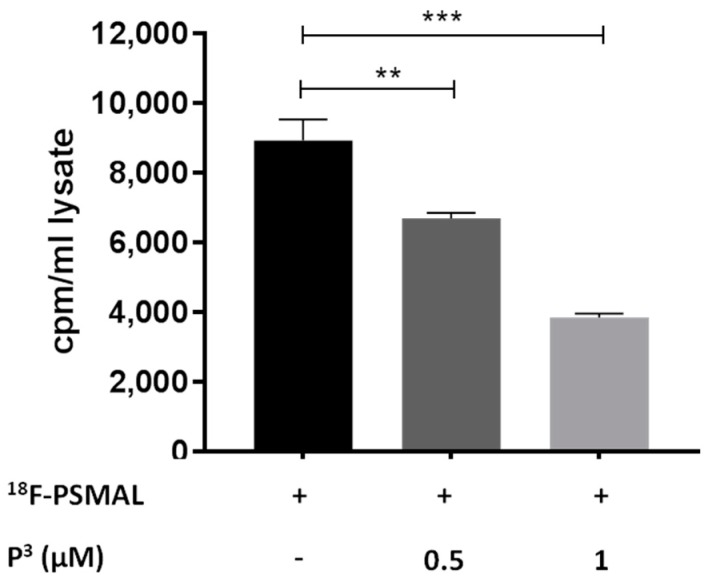
Uptake of ^18^F-PSMAL by LNCaP cells in presence of P^3^ (** *p* = 0.0088, *** *p* = 0.0001).

To enable insertion of PSMAL into the phospholipid bilayer of liposomes, we designed a compound P^3^ (Compound **9**) which is a molecule containing a lipophilic anchor linked to PSMAL via a polyethylene glycol (PEG_2000_) chain (Scheme 2). In P^3^ molecule, the long hydrocarbon chain of palmitate residue allows the molecule to interact with the phospholipid bilayer, whereas the PEG linker projects the Lys–urea–Glu motif on the surface of liposomes for binding to PSMA. We examined the binding of P^3^ to PSMA by using P^3^ as a competitor of ^18^F-PSMAL in a binding assay and by hypothesizing that P^3^ will compete with ^18^F-PSMAL at its receptor in LNCaP cells. As shown in Figure 5, P^3^ reduced the uptake of ^18^F-PSMAL in LNCaP cells in a dose-dependent manner, proving that the ability of the targeting motif to bind to PSMA protein was preserved in P^3^. 

### 3.4. P^3^ Could be Post-Inserted in Liposome Bilayer

**Figure 6:**
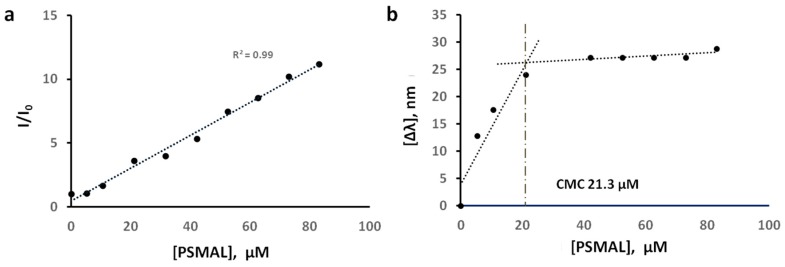
(**a**) Relative fluorescence intensity of *N*-phenyl-1-naphthylamine (NPN) in presence of varying concentrations of P^3^. (**b**) Shift in the emission maxima of NPN with respect to P^3^ concentration (λ_ex_ = 340 nm).

Conventionally, surface modification of liposomes is performed by combining the targeting reagent of interest with other lipids before liposome formation. This method results in liposomes displaying the targeting functionality on the outer as well as inner layer of the phospholipid membrane. Targeting motifs, such as P^3^, in the inner layer do not participate in the targeting process and are just wasteful. Apart from being inefficient, large PEG chains in the inner core of the liposomes occupy a significant internal volume of liposomes because of brush- or mushroom-like formations [21]. Consequently, drug-loading capacity of liposomes, retention of payload, and liposome stability are adversely affected [21,22,54,55]. Considering these issues, we functionalized phospholipid bilayer by post-insertion of lipopolymer P^3^ only on the outer surface of preformed liposomes. This method has been previously used successfully to engraft PEG derivatives in liposomes [21,22], and it includes incubation of liposomes with a micellar solution of the amphiphilic lipopolymer. Lipopolymer insertion in liposome bilayer is dependent on its CMC value [21,22]. At concentrations higher than their CMC, amphiphilic lipopolymers are not available as monomers as they aggregate to form micelles, creating a thermodynamic barrier against the post-insertion process. We determined CMC of P^3^ by using NPN as a membrane fluidity probe. *N*-phenyl-1-naphthylamine has weak fluorescence in aqueous solutions, whereas its fluorescence increases significantly when it is part of a micellar formation of amphiphilic compounds [26,56]. Thus, the relative fluorescence intensity of NPN (I/I_0_) and shift in wavelength of fluorescence emission at constant NPN concentration and variable P^3^ concentration provide a profile indicative of the CMC value (Figure 6). As shown in Figure 6a, the increase in P^3^ concentration resulted in a corresponding increase in I/I_0_, which suggests that lipopolymer P^3^ is forming micelles in which NPN is being incorporated. Next, we recorded the maximum wavelength of emitted light at 340 nm excitation wavelength; the shift in the wavelengths of emitted light with respect to P^3^ concentration (Δλ = λ_0_ − λ_P_^3^) is shown in Figure 6b. The shift-profile showed an inflection point at 21.3 µM (a concentration of P^3^ at which sudden change in Δλ occurs). Compared to P^3^, the CMC value of another commonly used lipopolymer distearoyl phosphatidylethanolamine-PEG_2000_ was determined to be 2.57 µM.

Using the CMC knowledge from above, we performed insertion of P^3^ in preformed liposomes by incubating the liposomes with P^3^ at concentration below its CMC. Below CMC, P^3^ was essentially present as non-micellar monomer, which maximized P^3^ insertion in lipid bilayer. Physicochemical characteristics of the resultant liposomes are provided in Table 1. Compared to Plain-liposomes, a slight increase in particle size was observed after post-insertion (*p* < 0.0001), possibly due to the presence of long PEG_2000_ chain and resultant increase in hydrated volume of post-inserted liposomes (P^3^-liposomes). The net negative charge of the liposomes was also significantly reduced in P^3^-liposomes (*p* = 0.0088). This decrease in charge is hypothesized to be due to the shielding effect of PEG chain on the surface charge of negatively-charged phospholipids [55,57,58]. 

Liposomes have been modified by post-insertion of PEG-containing lipopolymers [44]. This useful technique, also known as post-modification of liposomes, is beginning to attract applications in liposome-based drug and biologic delivery [21,55,59]. More recently, the effect of post-insertion of different PEG-derivatives into pre-formed liposomes on several physiochemical characteristics was investigated by Mare et al. [60]. This technique was first reported by Uster et al. for the insertion of PEG-derivatized phospholipid to confer stealth properties to the liposomes [61]. The insertion of lipopolymer is a spontaneous process, which is driven mainly by the hydrophobic interaction of membrane lipids and the hydrophobic part of lipopolymers. By its very nature, the process of post-insertion is most efficient when constituent phospholipids exhibit fluidity, close to its melting temperature (T_m_). However, to prevent leakage of intravesicular contents and vesicle fusion, we performed post-insertion without raising temperature to match T_m_ of DPPC. Although considered in gel state at temperature < T_m_, DPPC bilayers demonstrate significant mobility at temperatures well below its T_m_ of 41 °C [62,63]. Electron paramagnetic resonance spectra of spin-label 5-DOXYL in DPPC liposomes has been shown to carry a remnant of peak at 300 K (~26.5 °C) which characterizes mobility of the bilayer [64]. These studies suggest that our liposome bilayer was sufficiently fluid to enable interaction and subsequent insertion of P^3^ at working temperatures of ~27 °C. In addition, α-tocopherol, a constituent in our liposomes, is known to increase the fluidity of DPPC liposomes [65]. It has been shown that performing the process at elevated temperatures increases the insertion rate of some ligands [61]. However, post-insertion of an antibody-PEG_3400_-phosphatidylethanolamine conjugate in Doxil formulation (T_m_ = 51 °C) at 4 °C has been successfully reported elsewhere [66].

### 3.5. P^3^-Liposomes Bind to Purified PSMA Protein as well as Target PSMA^+^ LNCaP Cells

**Figure 7:**
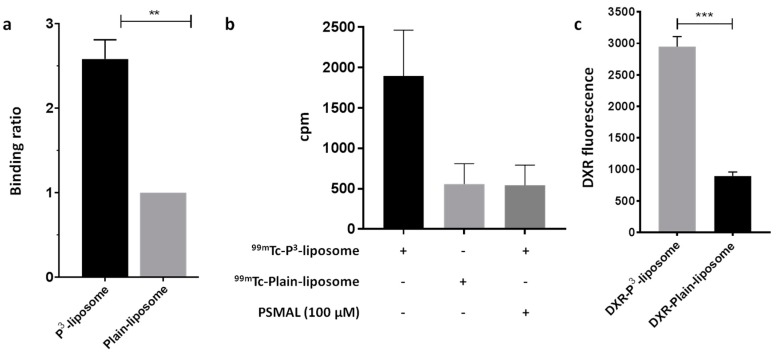
(**a**) Binding of ^99m^Tc-P^3^-liposomes and ^99m^Tc-Plain-liposomes to PSMA-column. (**b**) Interaction of ^99m^Tc-P^3^-liposomes and ^99m^Tc-Plain-liposomes with LNCaP cells in the presence and absence of unlabeled P^3^. (**c**) Delivery of doxorubicin by P^3^-liposomes and Plain-liposomes in LNCaP cells (** *p* = 0.0022, *** *p* = 0.0003).

In order to test whether P^3^-liposomes retain the ability to interact with PSMA protein, we used immobilized Ni-charged NTA to capture His-PSMA protein and allowed liposomes to interact with the resultant resin-bound PSMA protein. Both Plain- and P^3^-liposomes were labeled with ^99m^Tc to enable detection of bound liposomes. As shown in Table 1, ^99m^Tc-labeling efficiencies of the two liposome preparations were almost identical. These liposomes were allowed to pass through the PSMA column. Compared to ^99m^Tc-Plain-liposomes, ^99m^Tc-P^3^-liposomes were retained on the column significantly more, showing that the P^3^ moiety on the liposome surface was able to interact with PSMA protein bound to the column (Figure 7a). 

Next, we incubated PSMA^+^ LNCaP cells with ^99m^Tc-P^3^-liposomes or ^99m^Tc-Plain liposomes. After 1 h of incubation, the radioactive counts in cell lysates were determined. We found more than 3-fold higher radioactivity in the cells treated with ^99m^Tc-P^3^-liposomes as compared to the cells treated with ^99m^Tc-Plain-liposomes (Figure 7b). This uptake of ^99m^Tc-P^3^-liposomes was reduced in presence of unlabeled free P^3^ (Compound **9**) in the competition assay, which suggests that the uptake of P^3^-liposomes was mediated through binding to PSMA expressed on LNCaP cells (Figure 7b). In a parallel technique, we also investigated the targeting capability of P^3^-liposomes by loading them with doxorubicin and estimating the amount of intracellular doxorubicin in LNCaP cells after incubating the cells for 30 min. As shown in Figure 7c, P^3^-liposomes delivered more doxorubicin than the Plain-liposomes in LNCaP cells. 

Together, these data from three different techniques confirmed that liposomes post-inserted with P^3^ successfully target PSMA in cells and are capable of delivering therapeutics/diagnostics to prostate cancer cells which overexpress PSMA protein.

### 3.6. Doxorubicin-Loaded P^3^-Liposomes are More Cytotoxic than Doxorubicin-Loaded Plain-Liposomes in LNCap Cells

We investigated whether P^3^-liposomes are able to deliver cytotoxic concentration of doxorubicin in LNCaP cells as compared to Plain-liposomes. Although doxorubicin is not a drug of first choice for prostate cancer, it has been used by several groups to test targeting strategies using nanocarriers [46,51,67]. Easy loading in liposomes using standard ammonium-gradient liposomes, cost, and ability to colorimetrically track doxorubicin are a few reasons behind its widespread use. We prepared ammonium sulfate gradient liposomes carrying P^3^ in their bilayer structure. These liposomes were loaded with doxorubicin and compared with identical liposomes without P^3^ functionalization. Characteristics of these liposome preparations are given in Table 2. 

**Figure 8:**
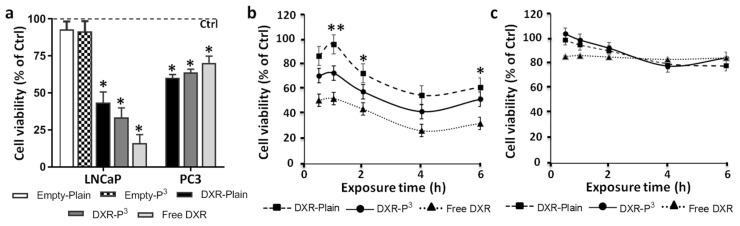
(**a**) Viability of LNCaP and PC3 cells after exposure to doxorubicin-Plain-liposome, doxorubicin-P^3^-liposomes, free doxorubicin, and empty liposomes for 24 h. (**b**,**c**) Viability vs. exposure time plots for LNCaP and PC3 cells, respectively, exposed to doxorubicin-Plain-liposome, doxorubicin-P^3^-liposomes, and free doxorubicin ((doxorubicin) = 1 µM, * *p* < 0.05, ** *p* < 0.01).

In initial experiments, LNCaP and PC3 cells were treated with doxorubicin-P^3^-liposomes and doxorubicin-Plain-liposomes for 48 h, with doxorubicin concentration maintained at 1 µM; as control, LNCaP and PC3 cells were also exposed to free doxorubicin in a similar manner. As additional control, we also tested cytotoxicity of empty (drug-free) Plain- and P^3^-liposomes in LNCaP cells; however, the empty liposomes were not found to have any effect on cell viability (Figure 8a). Determination of cell viability in LNCaP cells showed that although 48 h-exposure to doxorubicin-P^3^-liposomes was more toxic than 48 h-exposure to doxorubicin-Plain-liposomes, the difference was not significant; in addition, even PC3 cells showed significant toxicity to the 48 h-exposure to liposomal doxorubicin (Figure 8a). These observations suggested that prolonged exposure of cells to the drug nullified the targeting potential of P^3^-liposomes. In other words, P^3^-targeted as well untargeted doxorubicin-liposomes showed similar ability to become intracellular if exposure time was prolonged in cell culture. To overcome this issue, we exposed LNCaP and PC3 cells to doxorubicin-P^3^-liposomes and doxorubicin-Plain-liposomes for shorter durations of 0.5, 1, 2, 4, and 6 h before washing the cells with regular medium and allowing the cells to grow up to 48 h. This technique is closely mimics the exposure durations expected in vivo. As shown in Figure 8b,c, doxorubicin-P^3^-liposomes were clearly found to be more cytotoxic than doxorubicin-Plain-liposomes in LNCaP cells. Moreover, these shorter exposure times were found to be not significantly toxic in PC3 cells (Figure 8c). At the same time there was no difference in the cytotoxicity profile between doxorubicin-P^3^-liposomes and doxorubicin-Plain-liposomes in PC3 cells (Figure 8c). 

**Figure 9:**
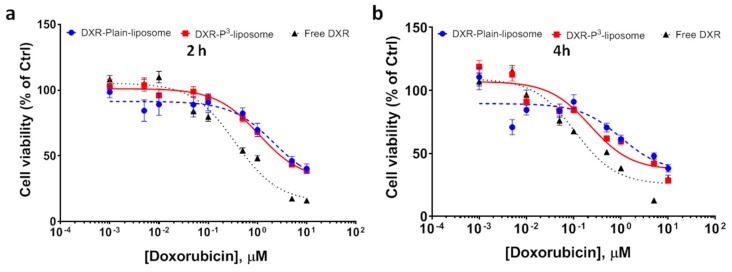
Dose–response curves of free doxorubicin, doxorubicin-P^3^-liposomes, and doxorubicin-Plain-liposomes in LNCaP cells. The cells were exposed to liposomes for (**a**) 2 h and (**b**) 4 h.

Data so far suggested that engrafting liposomes with P^3^ increased the toxicity of doxorubicin-loaded liposomes due to the PSMA-mediated targeting of P^3^-liposomes. To confirm, we determined IC_50_ of doxorubicin-P^3^-liposomes in LNCaP cells. The cells were exposed to various concentrations of liposomal doxorubicin for 2 h and 4 h and dose–response curves were obtained from the cell viability data (Figure 9). Analysis of dose–response curves by non-linear regression showed that IC_50_ values of doxorubicin-P^3^-liposomes were 1.06 µM for 2 h exposure time and 0.22 µM for 4 h exposure time. In comparison, doxorubicin-Plain-liposomes showed IC_50_ values of 2.16 µM and 1.0 µM, respectively. For free doxorubicin, the IC_50_ values for 2 and 4 h exposures were 354 nM and 107 nM, respectively. The IC_50_ values derived from non-linear regression of these dose–response curves were significantly different (*p* < 0.0001). 

## 4. Conclusions

The versatility of liposomes as a drug delivery vehicle have been reviewed on several occasions [68,69]. In this article, we report the synthesis of a P^3^ lipopolymer that could be used to functionalize liposomes for targeted delivery of therapeutics/diagnostics to PSMA^+^ prostate cancer cells. We post-inserted this lipopolymer on the surface of liposomes and showed that post-inserted liposomes are taken up by PSMA^+^ LNCaP cells. We demonstrated that liposomes loaded with doxorubicin and functionalized with PSMA-targeting ligand are more toxic to LNCaP cells than non-targeted liposomes. These findings suggest that liposomes carrying targeting lipopolymer P^3^ could be used for specific delivery of therapeutics/diagnostics to advanced/metastatic prostate cancer tumors. However as noted in the case of other PEG-modified liposomes, P^3^-liposomes can be influenced by accelerated blood clearance (ABC) phenomenon [70]. The ABC of PEG-liposomes is attributed to the formation of anti-PEG antibodies upon repeated administrations. It not only affects bioavailability, but also targeting and resultant efficacy of the liposome-encapsulated drug [71]. Thus, ABC phenomenon on repeated administration of P^3^-liposomes should be investigated, which can potentially impact their overall efficacy as a theranostic preparation. 

## Figures and Tables

**Table 1:** Physicochemical properties of Plain- and P^3^-liposomes.

Preparation	Hydrodynamic Diameter (±SEM) (nm)	Polydispersity (±SEM)	Charge (±SEM) (mV)^a^	(Phospholipid) (mg/mL)	^99m^Tc Labeling Efficiency (%)
Plain-Liposome	175.9 (±0.6574)	0.0423 (±0.0212)	−25.75 (±0.194)	60.36	90.30
P^3^-Liposome	187.37 (±0.1562)	0.0887 (±0.0119)	−13.87 (±2.481)	54.92	90.44

**Table 2:** Physicochemical properties of doxorubicin-loaded Plain-liposomes (doxorubicin-Plain-liposome) and doxorubicin-loaded P^3^-liposomes (doxorubicin-P^3^-liposome).

Preparation	Hydrodynamic Diameter (±SEM) (nm)	Polydispersity (±SEM)	Charge (±SEM) (mV)	(DOXORUBICIN) (±SEM) (µM)
Doxorubicin-Plain-liposome	183.84 (±0.89)	0.120 (±0.018)	−2.62 (±3.92)	215.55 (±8.45)
Doxorubicin-P^3^-liposome	184.05 (±0.87)	0.160 (±0.008)	−12.11 (±1.72)	248.89 (±6.94)
